# Sportsmans Groin: The Inguinal Ligament and the Lloyd Technique

**DOI:** 10.5334/jbr-btr.1404

**Published:** 2017-12-16

**Authors:** WJ Rennie, DM Lloyd

**Affiliations:** 1 Leicester Royal Infirmary, GB

**Keywords:** Groin pain, Inguinal Ligament, MRI, Surgery, Lloyd release

## Abstract

Groin pain is a catch all phrase used to define a common set of symptoms that affect many individuals. It is a common condition affecting sportsmen and women (1, 2) and is often referred to as the sportsman groin (SG). Multiple surgical operations have been developed to treat these symptoms yet no definitive imaging modalities exist to diagnose or predict prognosis. This article aims to discuss the anatomy of the groin, suggest a biomechanical pathophysiology and outline a logical surgical solution to treat the underlying pathology. A systematic clinical and imaging approach with inguinal ligament and pubic specific MRI assessment, can result in accurate selection for intervention. Close correlation with clinical examination and imaging in series is recommended to avoid misinterpretation of chronic changes in athletes.

## Introduction

Groin pain is a catch all phrase used to define a common set of symptoms that affect many individuals. It is a common condition affecting sportsmen and women [1,2] and is often referred to as the sportsman groin (SG). Multiple surgical operations have been developed to treat these symptoms, yet no definitive imaging modalities exist to diagnose or predict prognosis. This article aims to discuss the anatomy of the groin, suggest a biomechanical pathophysiology and outline a logical surgical solution to treat the underlying pathology.

## Definition of the Groin

The term groin encompasses an ill-defined area between the abdomen and upper thigh. This covers multiple anatomical structures and it is unsurprising that multiple procedures exist to deal with individual events that are involved in this region. The lack of specificity of the term also leads to much confusion [3]. The term sportsman hernia is also misleading and should be abandoned for more *organ specific* terminology rather than *regional based* terminology for a diagnosis. At a consensus meeting on sportsman’s groin it was concluded that the terminology to be used was *inguinal disruption*, while agreeing that palpable or visible disruption may not be obvious on presentation [11]. Specific diagnostic terminology which relates to anatomical structures should be adopted. This would include adductor longus tendonopathy, inguinal ligament strain or osteitis pubis for example. The pain from SG is due to altered biomechanics, with specific pain symptoms that differ from those caused by inguinal or femoral hernias.

## Anatomy of Sportsman’s Groin

**Figure 1:**
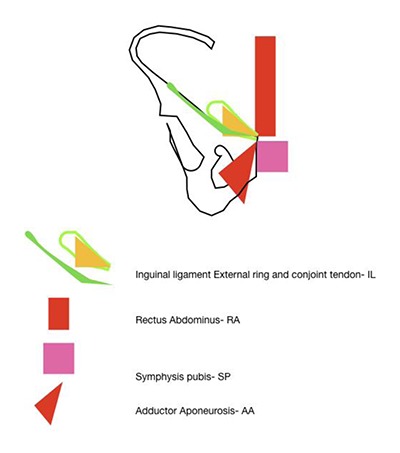
Structures attached to the pubic tubercle.

**Figure 2:**
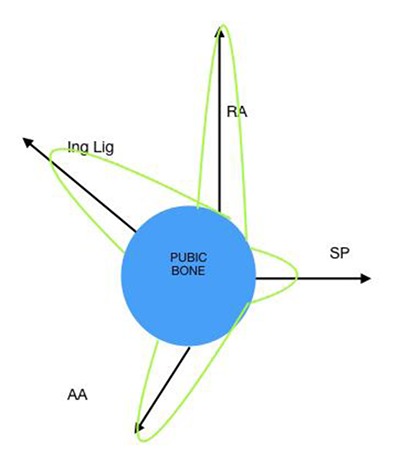
Vectors on the pubis as a clock-face.

The anatomical central structure in the groin is the pubic bone. Multiple muscular and ligamentous attachments exert forces onto this bone, which is part of the tri-radiate pelvis. The concept of balanced forces and a mechanical strain is a unifying theory of the confluence of anatomical structures on the pubis [4]. The broadest of these attachments is the inguinal ligament, along with the conjoined tendon and lacunar reflection. The common adductor insertion, the rectus abdominis attachment and the pubic symphysis all are attached at different points on the pubis (Figure 1). The various enthesal attachments can broadly be classified into separate vectors along a pubic clock-face and exert opposing pulling forces which in most cases are balanced. The schematic (Figure 2) demonstrates these vectors in varying directions from the enthesal attachments. The specific force exerted by the oblique abdominal wall muscles during twisting, turning and kicking maneuvers over a period of time, may cause tensioning and cordlike bands within the inguinal ligament.

## Clinical Features and Examination

Athletes present with pain around the pubic tubercle commonly with an insidious onset. Symptoms are exacerbated by kicking, twisting or sometimes by sneezing or by actions such as getting out of bed or a car. In many cases, the patient points to an area near the pubic tubercle. Clinical examination is performed by careful, systematic palpation of the structures around the pubic tubercle [3]. The adductor tendon, pectineus, lacunar ligament, inguinal ligament, conjoined tendon and rectus sheath attachment are examined – the so called pubic clock [5].

## Imaging Diagnosis

Specific magnetic resonance imaging (MRI) protocols have been reported as useful in the diagnosis of groin related pathology in athletes [14]. Recently, the use of MRI in the diagnosis of groin pain and adductor related pathology has been challenged [15,16], possibly due to the risk of misinterpretation of chronic imaging changes in asymptomatic athletes. These studies include changes in various anatomical structures and assess a multitude of MRI findings in the various groin anatomical structures. Inclusion of the hip and femoro-acetabular impingement is a major confounding factor in the diagnostic predictive value of MRI, sometimes leading to early and often unnecessary surgical intervention [18].

Very few studies have focused on the pubis and its attached structures, assessing the utility of clinical and MRI findings. A recent large prospective cohort study of athletes, using both specific clinical and MRI protocols and patient reported outcome measures, found the post-test odds ratio of clinical examination with MRI, conducted in series, increased as much as 6-fold [17]. Falvey, et al. termed this attachment the *pubic aponeurosis* (PA), which was described as a common confluence of the rectus abdominus and the adductor origin, without mention of the inguinal ligament. Using this definition, the presence of pubic bone marrow oedema was strongly related to the site of pain in their large cohort (p < 0.001).

No study so far focused specifically on the inguinal ligament and its relationship to site-specific bone marrow oedema. The imaging characteristics of the medial portion of the inguinal ligament and its pubic enthesal attachment were not specifically assessed.

**Figure 3:**
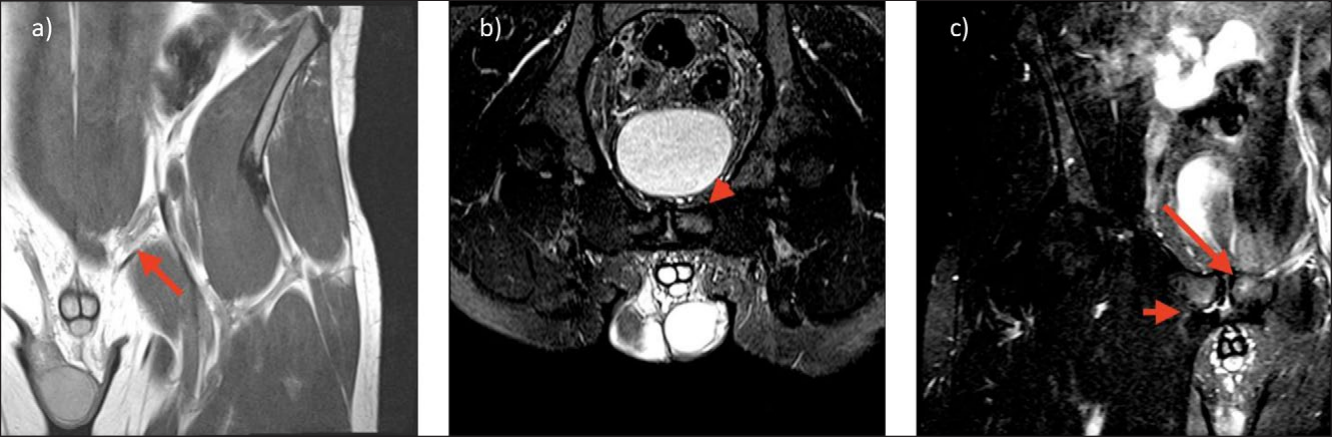
**a)** 30 year old footballer with left groin pain. Coronal oblique high resolution image through the medial aspect of the inguinal ligament adjacent to the pubic enthesis demonstrates a cord-like medial aspect of the inguinal ligament (red arrow). **b)** 30 year old footballer with left groin pain. Angled oblique axial inlet High resolution T2 fat suppressed images through the superior aspect of the pubic bone at the site of the inguinal ligament insertion demonstrates BMO of the left pubic bone (red arrowhead) **c)** 30 year old footballer with left groin pain. Coronal oblique High resolution T2 fat suppressed images through the adductor insertion on the right and pubic tubercle on the left, demonstrates site specific BMO of the left pubic bone at the inguinal entheses (long red arrow) and a right adductor cleft with associated site specific BMO (short red arrow).

Specific findings with inguinal ligament pathology are the morphology of the medial entheses of the inguinal ligament and site-specific bone marrow oedema of the pubis A band or cord like inguinal ligament maybe seen at surgery or on MRI imaging (Figure 3a, b, c).

## Biomechanics and Surgical techniques

In sports with lower limb centric rotational movements, specifically football with repeated pivoting forces with the lower limb in external rotation and extension, the vectors on the pubis may be out of balance. In this position, the inguinal ligament along with the pubic synchondrosis, provides a stabilising force, when significant pulls by the adductor and secondarily the rectus abdominus occur, when kicking the ball. Adductor tendinopathy is a commonly recognised injury as a part of the sportsman groin. Gilmore, initially popularised the concept of pain in this region as the Gilmore’s groin. Whereas, his repair focused mainly on a torn external oblique, conjoined tendon, as many as 40% of sportsmen may have an adductor or rectus tear [6]. Procedures that repair the attachments in this region with reattachment of the adductor tendon attachment, have been proposed by Meyer, et al. after diagnosis using a combination of clinical and MRI findings [7].

Broadly, the many surgical techniques that exist may be grouped into tensioning [8,12,13] or de-tensioning [4,5,6,7,8,9] of the inguinal ligament or its surrounding soft tissues.

The inguinal ligament release technique described by Lloyd, et al. in 2008, was developed to specifically treat athletes with tenderness on the pubic tubercle at the attachment of the inguinal ligament. The procedure is performed laparoscopically whereby the inguinal ligament is divided and released from the pubic bone and the groin reinforced with a mesh. The operation is similar to a standard laparoscopic inguinal hernia repair. In addition, any scar tissue or sutures from previous surgery are removed, any thickening of the pectineus fascia is released and if thickened, the lateral few millimetres of the rectus sheath insertion, divided [9]. Studies have reported more than 90% of athletes return to full fitness levels, at a four-week follow up following the procedure [10].

## Differential Diagnosis

MRI is useful in the assessment of structures that produce groin related symptoms. In the hip, psoas bursitis and tendinopathy can present with groin related symptoms. MRI can demonstrate hyperintense T2 signal within the tendon at its insertion, mucoid changes in the bursa or feud within the bursa. Trochanteric bursitis can be differentiated with Ultrasound and MRI as a cause of symptoms which can sometimes be more central in location. Athletes involved in throwing sport can also present with central midline pain at the pubis related to strain of the rectus abdominus or related to strains of the oblique muscles of the abdomen-a side strain injury. This can be assessed on high resolution MRI imaging and graded accordingly.

It is important to distinguish hip articular and bone related pathology such as stress fractures and femeroacetabular impingement. These can be assessed using a combination of radiographs and MRI. Postoperatively, neuromas of the ilioinguinal and genitofemoral nerves, seromas and mesh related pathology can be assessed using ultrasound. The post-operative integrity of the mesh can be dynamically assessed using ultrasound (US). Infection though rare primarily, as a cause of groin symptoms, can occur after interventions such as injections or postoperatively and imaging with MRI and US are helpful in its detection.

## Conclusion

SG Pain is a common condition affecting more than 5% of athletes involved in kicking or twisting sports. The abdominal core muscles play a pivotal role in the cause of SG as their forces are transmitted through the inguinal ligament onto the pubic tubercle. The inguinal ligament release technique with additional mesh repair is a procedure specifically developed to treat the target lesion and allow early return to sport. A systematic clinical and imaging approach with inguinal ligament and pubic specific MRI assessment, can result in accurate selection for intervention. Close correlation with clinical examination and imaging in series is recommended to avoid misinterpretation of chronic changes in athletes.
